# The Impact of a State-Sponsored Mass Media Campaign on Use of Telephone Quitline and Web-Based Cessation Services

**DOI:** 10.5888/pcd11.140354

**Published:** 2014-12-24

**Authors:** Jennifer C. Duke, Nathan Mann, Kevin C. Davis, Anna MacMonegle, Jane Allen, Lauren Porter

**Affiliations:** Author Affiliations: Nathan Mann, Kevin C. Davis, Anna MacMonegle, Jane Allen, RTI International, Research Triangle Park, North Carolina; Lauren Porter, Florida Department of Health, Tallahassee, Florida.

## Abstract

**Introduction:**

Most US smokers do not use evidence-based interventions as part of their quit attempts. Quitlines and Web-based treatments may contribute to reductions in population-level tobacco use if successfully promoted. Currently, few states implement sustained media campaigns to promote services and increase adult smoking cessation. This study examines the effects of Florida’s tobacco cessation media campaign and a nationally funded media campaign on telephone quitline and Web-based registrations for cessation services from November 2010 through September 2013.

**Methods:**

We conducted multivariable analyses of weekly media-market–level target rating points (TRPs) and weekly registrations for cessation services through the Florida Quitline (1-877-U-CAN-NOW) or its Web-based cessation service, Web Coach (www.quitnow.net/florida).

**Results:**

During 35 months, 141,221 tobacco users registered for cessation services through the Florida Quitline, and 53,513 registered through Web Coach. An increase in 100 weekly TRPs was associated with an increase of 7 weekly Florida Quitline registrants (β = 6.8, *P* < .001) and 2 Web Coach registrants (β = 1.7, *P* = .003) in an average media market. An increase in TRPs affected registrants from multiple demographic subgroups similarly. When state and national media campaigns aired simultaneously, approximately one-fifth of Florida’s Quitline registrants came from the nationally advertised portal (1-800-QUIT-NOW).

**Conclusion:**

Sustained, state-sponsored media can increase the number of registrants to telephone quitlines and Web-based cessation services. Federally funded media campaigns can further increase the reach of state-sponsored cessation services.

## Introduction

Smoking cessation treatment offered by telephone quitlines is a core element of comprehensive US state tobacco control programs (1). Through quitlines, free telephone counseling services are available in all 50 states, the District of Columbia, Guam, and Puerto Rico (2). Web-based interventions also provide additional assistance to smokers in 24 states. Emerging evidence supports the potential efficacy of Web-based interventions for adult smoking cessation (1,3,4).

Although millions of US smokers attempt to quit smoking each year, only 3% to 5% of smokers remain quit 6 to 12 months later (5). The low success rate for smokers’ quit attempts is due, in part, to the low proportion (22%) of smokers who use evidence-based interventions during quit attempts (6). Currently, quitlines are used by only 1% to 2% of US smokers (7), and the potential role of quitlines and Web-based treatments in reducing population-level tobacco use is contingent on successfully promoting greater use.

Television advertising has been widely used to promote telephone quitlines (8), and there is strong evidence for its effectiveness in the United States (9,10) and internationally (10–15). A study of call volume to 9 US state quitlines found that increases in exposure to televised tobacco countermarketing advertisements (measured in quarterly target rating points [TRPs]) during 3 years were associated with increases in call volume (16). To date, little is known about the extent to which US state-sponsored television advertising influences the use of quitlines or Web-based cessation services in the context of additional, intermittent advertising from a federally funded tobacco education campaign, the Centers for Disease Control and Prevention’s (CDC’s) Tips From Former Smokers (“Tips”). Studies document the effectiveness of quitline advertising among several population subgroups. Targeted advertising has increased call volume among pregnant women (17), African Americans (18), and indigenous populations in New Zealand (13). An Australian study found that the effect of antitobacco television media campaigns on call volume did not vary by socioeconomic status (19). No studies have examined the extent to which antitobacco advertising influences the demographic composition of quitline and Web-based cessation service users.

Research on the effect of mass media campaigns on Web-based cessation services is limited. A natural history time-series analysis of the 2012 national Tips campaign found that the number of unique visitors to the campaign’s cessation website, www.smokefree.gov, was more than 4 times the number of visitors during the same period in the previous year (20). No studies have examined the effect of state-sponsored televised mass media campaigns on the use of Web-based cessation services.

Our study evaluates the effects of a sustained tobacco cessation media campaign on registrations with the Florida Quitline and Web-based cessation services (Web Coach) during 35 months (November 2010 through September 2013). The primary objective was to examine the relationship between market-level measures of media campaign delivery, as measured by television TRPs, and the number of Florida Quitline and Web Coach registrants. Results from this analysis will be used to estimate the total number of smokers that registered for each type of cessation service as a result of the campaign. A secondary objective was to address a gap in the research literature on the advertising effects on demographic subpopulations by examining the extent to which shifts in media delivery influence the composition of registrations with the Florida Quitline and Web Coach.

## Methods

### The Tobacco Free Florida Campaign

Florida had the highest funding level of any state tobacco control program at $64.3 million in 2013 (21). The average per capita funding for Florida’s tobacco control program in 2013 ($3.37) was higher than the average for other states ($1.46). During the study period, Florida spent an average of $21 million annually on its media campaign, Tobacco Free Florida (TFF). Since November 2010, Alma DDB and partners have planned and implemented the campaign, which includes cessation messages for adult smokers in multiple media formats, including television (broadcast and cable), radio, social media (Facebook, Twitter, and YouTube), digital advertisements, and earned media and public relations. Both English and Spanish language advertisements are promoted. TFF campaign television advertisements targeted adult smokers with cessation messages during the study period. Advertisements airing from November 2010 through June 2012 consisted of graphic content and/or emotionally evocative personal testimonials that previously aired in New York, Massachusetts, and Australia and were shown to be effective (22–24). Advertisements airing from July 2012 through September 2013 consisted of Tips advertisements available through CDC’s Media Campaign Resource Center. Tips was found to increase quit attempts among US smokers (25). TFF advertisements were tagged with the state telephone number (1-877-U-CAN-NOW) and/or a cessation resource website (www.quitnow.net/florida), with both services advertised for 33 of the 35 study months. Tips television advertisements were tagged with the national portal telephone number (1-800-QUIT-NOW) and/or a cessation-resource website that provided the portal URL (Smokefree.gov).

### Data sources and measures

This study examined individual-level data from tobacco users who registered for cessation services through the Florida Quitline or Web Coach. Data from both sources included registrants’ demographics, geographic location, smoking status, and cessation behaviors. Registrants were defined as tobacco users who provide personal information and agree to receive services from the Quitline. This study examined weekly Quitline and Web-based registrants from November 2010 through September 2013. Data were collected by Alere Wellbeing, the provider for both services.

Market-level exposure to the media campaign was measured in Florida’s 10 media markets by using TRPs, a standard media-buying metric that measures the potential reach and frequency of exposure to television advertisements. TRPs are defined as the product of the percentage of the audience that is potentially exposed (ie, audience reach) and the frequency of that exposure (ie, the number of times advertisements were aired). For example, if 75% of a media market’s television audience was exposed to an advertisement twice per week, the number of weekly television TRPs in the market would equal 150 (75 × 2) (8). Data on television TRPs were provided weekly by the media contractor, Alma DDB. Free nicotine replacement therapy (NRT) giveaway programs occurring 19 weeks during the study period were also documented because advertising these programs increases call volume to quitlines (26–28).

### Analysis

We performed all statistical procedures using Stata 13 (StataCorp, LP), and statistical significance was reported at *P* < .05. We summarized descriptive data on the characteristics of Florida’s Quitline and Web Coach registrants and compared each by type. We compared registrants with the population of tobacco users residing in Florida, using data from the 2010 US Census and 2011 Florida Adult Tobacco Survey. We further examined the relationship between the weekly number of registrants to Quitline and Web-based services and weekly TRPs by media market. To demonstrate trends in registrants and media exposure, we plotted descriptive data.

We also conducted multivariate linear regression models estimating the level of Quitline registrations as a function of media market-level weekly TRPs. For each registrant, we examined the corresponding media market’s TRPs during the week in which they registered. Total weekly Quitline registrations were regressed on Florida TRPs, controlling for media-market–level variables, including the percentages of the media market population that were African American or Hispanic, the percentage of the media market population that had a bachelor’s degree or higher, and the median household income in the media market. A linear time trend was included to account for weekly changes in call volume over time, independent of the TFF campaign. In addition, an indicator variable was included as a control variable to account for the presence of a tagline advertising the availability of free NRT. Additional media exposure to the national Tips television advertisements — resulting from CDC’s media buy in Florida media markets — was measured using TRPs and included as a separate control variable in analyses. The same analyses were conducted with data from Web registrants during the study period, with one exception: we did not include media exposure to the national Tips television advertisements because they do not advertise the Florida state Web-based services.

Because both models control for NRT advertising, we conducted additional stratified analyses to determine whether the NRT variable affected the magnitude of TRP effects. The results were similar, suggesting no significant effect.

In addition to the overall models, we explored TRP effects in different demographic groups: white people and people who are not white; males and females; those younger than age 35 and those aged 35 or older; those with no more than a high school degree and those with at least some college; and those who smoked less than 20 cigarettes per day and those who smoked 20 or more cigarettes per day. To examine whether the composition of registrants changed as media exposure fluctuated, registrations for each group were compared by using the Florida population sizes for each demographic group.

Using regression model results, we then performed postestimation predictions under a counterfactual assumption of no antitobacco advertising (TRPs = 0) to estimate the total additional registrations that were attributable to television advertising in Florida. We also calculated the proportion of all Florida tobacco users who registered for cessation services in Florida in 2011 and 2012.

## Results

In total, 141,221 tobacco users registered for cessation services through the Florida Quitline, and 53,513 tobacco users registered for cessation services through Web Coach (Table 1). Compared with the proportion of tobacco users statewide, both Florida Quitline and Web-based services were used by a larger proportion of females, people with more than a high school education, and tobacco users aged 35 to 54. Registrants smoked more cigarettes per day and were more addicted than were tobacco users statewide. Whereas 47.5% of Florida Quitline registrants and 53.8% of Web Coach registrants were uninsured, only 32.2% of tobacco users statewide were uninsured. Compared with Web-based registrants, Quitline registrants were older, had a lower education level, and were more likely to be nonwhite.

**Table 1:** Demographic and Smoking-Related Characteristics of Florida Tobacco Users Who Registered for Cessation Services, November 2010 Through September 2013^a^

Characteristic	Florida Quitline (N = 141,221), No. (%)	Florida Web Coach (N = 53,513), No. %	Population of Florida Tobacco Users 2012, %
**Sex**			
Male	59,222 (43.5)	25,122 (47.0)	57.4^b^
Female	76,840 (56.5)	28,388 (53.0)	42.6^b^
**Age, y**			
18–24	11,926 (8.9)	5,813 (10.9)	12.6^b^
25–34	25,706 (19.3)	14,623 (27.3)	20.8^b^
35–54	63,645 (47.7)	25,396 (47.5)	40.1^b^
55 or older	32,064 (24.1)	7,677 (14.4)	26.6^b^
**Race/ethnicity**			
White non-Hispanic	86,390 (69.7)	40,338 (78.3)	66.8^b^
Black non-Hispanic	15,010 (12.1)	2,860 (5.6)	12.2^b^
Other non-Hispanic	2,162 (1.7)	1,555 (3.0)	4.1^b^
Hispanic	20,434 (16.5)	6,764 (13.1)	16.9^b^
**Education**			
High school graduate or less	65,764 (51.9)	20,148 (39.6)	58.3^b^
At least some college	60,943 (48.1)	30,732 (60.4)	41.7^b^
**Cigarettes per day**			
Mean number	17.7	18.3	12.4^c^
**Time from awakening to first cigarette**			
Less than 5 min	61,176 (47.9)	21,200 (40.4)	21.1^c^
5 min or more	66,452 (52.1)	31,276 (59.6)	78.9^c^
**Insurance status**			
Insured	68,429 (52.5)	23,756 (46.2)	67.8^b^
Not insured	61,813 (47.5)	27,681 (53.8)	32.2^b^

a Not all subcategories sum to total because of missing values.

b Data source: Behavioral Risk Factor Surveillance System (29).

c Data source: Florida Department of Health (30).

Variation in the number of weekly registrants for both services corresponded closely with patterns in the number of weekly television TRPs, and the number of registrations was higher for Quitline services than for Web-based services (Figure). The number of registrants for cessation-services was highest during the second quarter of 2012, when the campaign highlighted the availability of free NRT through multiple media channels and when CDC’s national Tips campaign aired. During the period of national Tips advertising, an estimated 18% to 23% of Florida Quitline registrants were transferred to the state telephone service from the national portal (1-800-QUIT-NOW).

**Figure:**
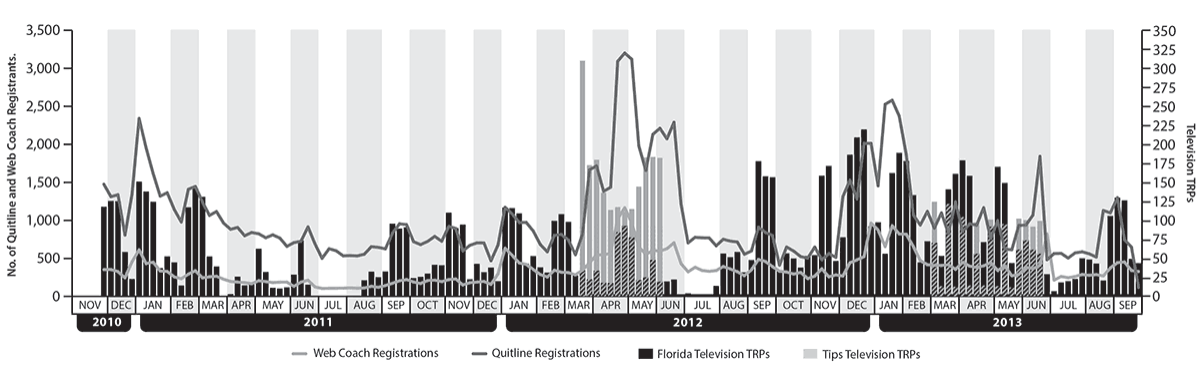
Tobacco Free Florida and Tips television target rating points (TRPs) and registrants for Florida Quitline and Web Coach, November 2010 through September 2013.

A regression model showed that the number of television TRPs was positively associated with the number of Florida Quitline registrants (Table 2). An increase in 100 TRPs per week in TFF television advertisements in a given market was associated with an increase of 7 registrants per week per media market (β = 6.8, *P* < .001). TRPs for advertisements highlighting free NRT resulted in more weekly registrants than TRPs with no mention of free NRT (β = 31.8, *P* < .001). Additional regression models for each demographic and smoking-related subgroup indicated that an increase in TRPs resulted in significantly more weekly Florida Quitline registrants for all subgroups examined. The demographic composition of Florida Quitline registrants did not change significantly as the number of TRPs increased.

**Table 2:** Regression of Florida Quitline and Web Coach Registrants on Target Rating Points (TRPs) for Tobacco Free Florida (TFF) Television Advertisements in Florida, November 2010 Through September 2013^a^

Independent Variable	Quitline Beta (95% CI) [*P* Value]	Web Coach Beta (95% CI) [*P* Value]
TFF television TRPs	6.8 (3.9 to 9.6) [<.001]	1.7 (0.6 to 2.8) [.003]
“Tips” television TRPs	11.4 (8.3 to 14.4) [<.001	—
Media market population size (in 100,000s)	−1.9 (−2.2 to −1.6) [<.001]	−1.1 (−1.2 to −1.0) [<.001]
Percentage of media market population that is black	−0.3 (−0.5 to −0.2) [<.001]	−0.2 (−0.3 to −0.2) [<.001]
Percentage of media market population that is non-white Hispanic	2.0 (1.6 to 2.1) [<.001]	0.8 (0.7 to 0.9) [<.001]
Percentage of media market population that has a bachelor’s degree or higher	−2.0 (−2.4 to −1.7) [<.001]	−1.0 (−1.2 to −0.8) [<.001]
Media market average income (in 10,000s)	19.8 (14.5 to 25.1) [<.001]	11.3 (9.2 to 13.4) [<.001]
Presence of advertisement highlighting free nicotine replacement therapy	31.8 (26.8 to 36.8) [<.001]	15.3 (13.3 to 17.3) [<.001]

Abbreviation: CI, confidence interval.

a Models include a control variable for a weekly time trend.

A second regression model showed that the number of television TRPs was also positively associated with the number of Web Coach registrants (Table 2). An increase in 100 TRPs per week in TFF television advertisements in a given market was associated with an increase of 2 registrants per week (β = 1.7, *P* = .003). As in the Florida Quitline model, advertisements highlighting free NRT availability resulted in more weekly registrants than advertisements not mentioning free NRT (*P* < .001). Additional regression models for Web Coach supported the findings by demographic and smoking-related subgroups for the Florida Quitline. Increases in the number of TRPs resulted in more weekly Web Coach registrants for all subgroups, but they did not alter the demographic composition of the registrants.

Postestimation predictions of Florida Quitline and Web Coach registrants in the absence of antitobacco television advertising showed that 22,102 tobacco users registered for Florida Quitline services and an additional 3,211 tobacco users registered for Web Coach as a result of antitobacco television advertising. In 2011, 1.6% of Florida tobacco users registered for cessation services in Florida: 1.3% through the Florida Quitline and 0.3% through Web Coach. In 2012, 2.6% of Florida tobacco users registered for cessation services in Florida: 1.8% through the Florida Quitline and 0.8% through Web Coach.

## Discussion

Study findings indicated that a sustained, state-sponsored media campaign featuring graphic and emotionally evocative personal testimonials increased the number of registrants to telephone quitlines and Web-based cessation services. The results indicated a dose effect: higher TRP levels resulted in more tobacco users registering for both types of cessation services, with television TRPs driving more Florida Quitline than Web Coach registrations. Although previous studies arrived at similar findings for media dose and quitline call volume (2,8,16), our study is the first to expand these findings to Web-based cessation services. In addition, our study found that advertising increased service utilization among all demographic subpopulations in Florida and that the composition of the Florida Quitline and Web-based tobacco users did not change when levels of advertising increased. These data suggest that a broadly targeted media campaign increased the use of cessation services among all Floridians. Our findings also suggest that the national Tips campaign further increased the use of Florida’s cessation services and nationally advertised services (2).

This study is timely and relevant given a new emphasis, across all public service sectors of the government, on using new technologies strategically to engage with citizens (31). Approximately half of all states offer interactive Web-based cessation counseling, yet few studies have calculated their reach or their utilization in their state population. This study showed that media advertisements increased the use of state-sponsored Web-based cessation services that have been shown to be effective (32).

This study did not examine cessation as a result of registrations, and quit rates associated with each of Florida’s 2 types of services are not directly comparable because of differences in the demographic characteristics of users and rates of attrition (33). However, it is notable that media exposure to antitobacco television advertisements, even without specific taglines for the services, led Florida tobacco users to seek out and use free Web-based cessation services. Our findings support conclusions from systematic reviews that the efficacy of Web-based interventions seems promising (3,4). Design and delivery of Web-based cessation programs are diverse (3,4), and they provide a potentially cost-effective way to reach larger numbers of smokers than do telephone quitline services alone (34). Free Web-based services, available at any time, may increase the reach of cessation interventions to smokers, allowing them to respond to advertisements for cessation assistance when quitline services are not available (eg, evening and weekends). Future research on the efficacy and cost-effectiveness of Web-based services is warranted.

Because media campaigns can have immediate effects on quitline use (8,9), sustained exposure to antitobacco messages is required to maintain consistently high utilization (14). Florida’s TFF campaign aired cessation-targeted television advertising at high levels and was augmented by federally sponsored advertising during the study period. Population reach for Florida’s Quitline and Web-based services ranged from 1.8% in 2011 to 2.2% of tobacco users in 2012, similar to 2011 utilization rates in other states with large populations, such as New York (1.7%) and California (0.8%). Although a study in Maine found that 6% of state tobacco users used cessation services in 2005 (35), more recent data show that 2.3% used cessation services in 2011 (36). Taken together, these data suggest that population reach for quitlines may range from 1% to 2%, even in states with comprehensive tobacco control programs that include adult-focused media campaigns. Future research investigating state utilization rates in the context of 2013 and 2014 national Tips advertising may add further insight on the potential population reach of cessation services.

Our study has several limitations. First, data are for cessation-service registrants. Not all registrants received a minimal dose of cessation services, so the treatment reach of the services may be lower than registration reach (eg, data indicate that 90.2% of tobacco users who use the services quit for at least 24 hours). However, most studies examine fluctuation in media and call-volume data (total number of attempted calls), which overestimate service use (eg, cessation-services registrants in Florida are 30% of the total call volume in Florida). Second, other unmeasured factors may affect cessation service utilization, such as noncampaign–specific promotions of cessation resources, recommendations from health care providers or others, and recurring use by past registrants. However, these effects probably occurred at consistent levels, based on Florida’s programmatic efforts during the study period. Third, potential exposure to other types of advertising (ie, radio, Internet, and print) that may have influenced registrants was not analyzed because of the low levels of advertising (ie, all other sources accounted for less than 25% of the media budget for the study period) and the lack of variation over time (eg, statewide Internet media aired at consistent levels with little fluctuation during the study period). Further research is warranted on the role of Internet media and other channels of cessation service utilization, especially Web-based services.

These findings have important implications for research and practice. Our results confirm that national media campaigns can increase the reach of cessation services in the context of a well-funded, state-sponsored televised mass media campaign. States should continue to promote quitlines as effective, population-based interventions that increase successful quitting (2,20). States that offer or expand the use of evidence-based services online may further increase the reach of services. The study suggests that, in practice, federally sponsored campaigns can augment state-based advertising of cessation services and help states maximize population-level tobacco cessation.
